# Ultrasound biomicroscopy assessment of the subclinical involvement of the aqueous drainage structures after silicone oil tamponade

**DOI:** 10.1007/s10792-025-03464-y

**Published:** 2025-04-04

**Authors:** Paolo Mora, Carlo Bellucci, Maurizio Rossi, Fernando O. Avellis, Benjamin D. Shkurko, Alessandra Romano, Salvatore A. Tedesco, Stefano Gandolfi

**Affiliations:** 1 Ophthalmology Unit, University Hospital of Parma, Parma, Italy; 2 Department of Clinical and Experimental Medicine, University Hospital of Parma, Parma, Italy; 3 Ophthalmology Unit, Department of Medicine and Surgery, University of Parma, Via Gramsci 14, 43126 Parma, Italy

**Keywords:** Rhegmatogenous retinal detachment, Silicone oil tamponade, Ultrasound biomicroscopy, Secondary glaucoma, Vitreoretinal surgery, Ocular echography

## Abstract

**Purpose:**

To examine the subclinical ultrasound biomicroscopy (UBM) changes in the anterior segment (AS) of eyes after silicone oil tamponade (SOT) permanence.

**Methods:**

This observational study enrolled patients undergone pars plana vitrectomy for retinal detachment with SOT. UBM was performed shortly before removing the SOT from eyes without any clinically detectable emulsion/particle in the AS (i.e., the study cohort). For each subject, four radial scans of the AS were obtained circumferentially for both the study and the fellow non-operated eye (i.e., the control cohort). A region of interest (ROI) was identified in each UBM scan and the maximum, minimum, and mean echo-reflectivity were assessed using imageJ software.

**Results:**

The study enrolled 33 patients (66 eyes). Of the study eyes, 15 underwent high-density tamponade (SOX), 11 underwent 2000cSt tamponade (SO2), and seven underwent 5000cSt tamponade (SO5). Significantly higher echo-reflectivity was detected in the superior quadrants of groups SO2 and SO5 (*P* = 0.04 and 0.02, respectively) and in the inferior quadrant of group SOX (*P* < 0.01) compared to the corresponding control eyes. In group SO2, the duration of the intraocular tamponade positively correlated with the number of hyper-reflective quadrants (*P* < 0.01).

**Conclusions:**

The significant relationship between SOT density and the localization of areas of increased echo-reflectivity supports the actual relationship between SOT trabecular impregnation and UBM findings, even in eyes free from visible emulsions. In the SO2 group, the duration of the intraocular SOT correlated with the extent of the involvement of the filtering structures.

## Introduction

Silicone oil tamponade (SOT) is part of the surgical management of complex retinal detachment (RD) as it is effective when air/gas tamponade is deemed inadequate [1–3]. Commercial SOT preparations are mixtures of linear polydimethylsiloxane polymers with different chain lengths and perfluorohexyloctane components, with compounds with various molecular weights and viscosities. Because they are transparent, inert, and have high interfacial tension with water, SOT is widely used for mid- to long-term intraocular permanence. Although the clinical benefits of SOT are evident, adverse effects and SOT-related ocular pathology are known [4–6]. These are related to the dispersion of emulsified SOT from the vitreous chamber toward different ocular compartments [7]. In particular, flooding of the aqueous drainage pathways by macro- and microparticles of emulsified SOT in the anterior chamber of the eye can lead to secondary glaucoma [8, 9]. A potential toxicity of unlabeled contaminants present in the commercial formulations has also been suggested [10].

Ultrasound biomicroscopy (UBM) uses frequencies of 35–100 MHz to visualize intraocular structures with a resolution of up to 20 μm axially and 50 μm laterally. This resolution can detect very subtle changes in the anterior segment (AS) structures, including those beyond the limit of penetration of imaging techniques such as optical coherence tomography. The UBM features of SOT emulsifications in the AS have been described extensively [11–15], while fewer studies have compared different types of SOT and possible UBM changes in eyes without clinical evidence of SOT emulsification.

This study enrolled pseudophakic eyes that underwent SOT to treat RD. Shortly before the scheduled removal of the tamponade fluid, the iridocorneal angle structures of eyes with no clinical evidence of emulsification in the AS were assessed using UBM. Three different types of SOT were evaluated and possible correlations between ultrasound anomalies and the densities of the three SOT formulations and the duration of intraocular SOT were analyzed.

## Methods

This single-center observational study included eyes that underwent SOT following pars plana vitrectomy (PPV) for RD. The type of SOT was chosen intraoperatively by the surgeons (PM and SAT) based on the RD characteristics: Siluron 2000® (Fluoron, Ulm, Germany), a mixture of 5% polydimethylsiloxane (PDMS) with a shear viscosity of 2,500,000 cSt, and 95% PDMS with a shear viscosity of 1,000 cSt (group SO2); RS-OIL ECS5000® (Alchimia, Ponte San Nicolò, Italy), consisting of 100% PDMS with a kinematic viscosity of 5,000 cSt (group SO5); and Densiron® XTRA (Fluoron, Ulm, Germany) consisting of a mixture of ultra-purified PDMS (69.5%), 10% very high molecular weight silicone oil, and ultra-purified perfluorohexyloctane (group SOX). The study was conducted in the Ophthalmology Unit of the University Hospital of Parma, Italy, in accordance with the Declaration of Helsinki with the approval of the local Ethics Committee (#1396/2020). Informed consent was obtained from the enrolled subjects. We included patients between 18 and 75 years old scheduled for tamponade fluid removal after prior uneventful PPV to treat rhegmatogenous RD. In all the SO-filled eyes (*i*.*e*., the study eyes), removal was planned according to the RD clinical course, respecting the recommended delay for each type of tamponade, in increasing order: within 90, 120, and 180 days for SOX, SO2, and SO5, respectively. The delay varied depending on the patient’s clinical condition and some limitations experienced during the COVID-19 pandemic.

To be included, patients were required to have no history of glaucoma medication/surgery before the RD. Starting from tamponade placement, they might receive hypotensive medications when necessary to maintain the intra-ocular pressure (IOP) ≤ 24 mm. The fellow eyes of each operated one, if never subject to PPV or glaucoma surgery, formed the control cohort. The study visit occurred during the SOT removal preoperative examination, around 1 week before the second PPV. The assessment included measuring the visual acuity and IOP with Goldmann’s applanation tonometry and a slit lamp examination with gonioscopy specifically to exclude signs of inflammation (peripheral anterior synechiae, the Tyndall effect, and corneal deposits) and evidence of SOT emulsification bubbles in the AS structures. We excluded eyes with any large SOT bubbles or hyperoleon, and those with a score > 0 at gonioscopy according to the SOT intraocular emulsion grading system [15].

### UBM assessment

**Fig. 1:**
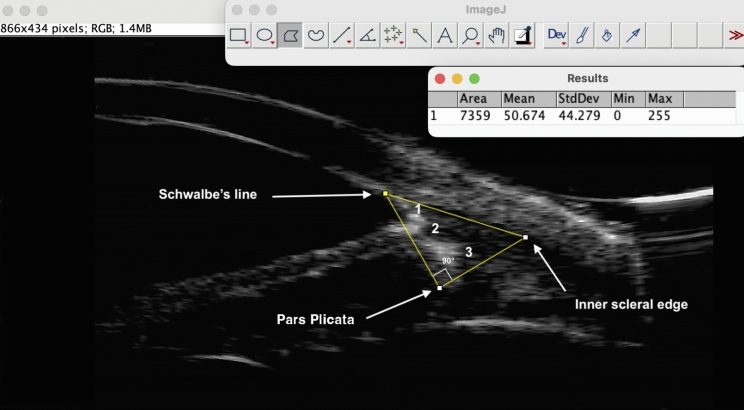
Example of how a Region of Interest (ROI) was created in a study eye using ImageJ. As a starting point, the Schwalbe’s line (SL) was identified. The first side of the right triangle was traced from the SL to the medial apex of the pars plicata (PP). The second side was traced perpendicularly from PP to the inner scleral edge. From this point the third side went back to the SL. Areas of increased echo-reflectivity can be observed in the trabecular meshwork (1), the iris root (2) and the ciliary body (3)

**Fig. 2:**
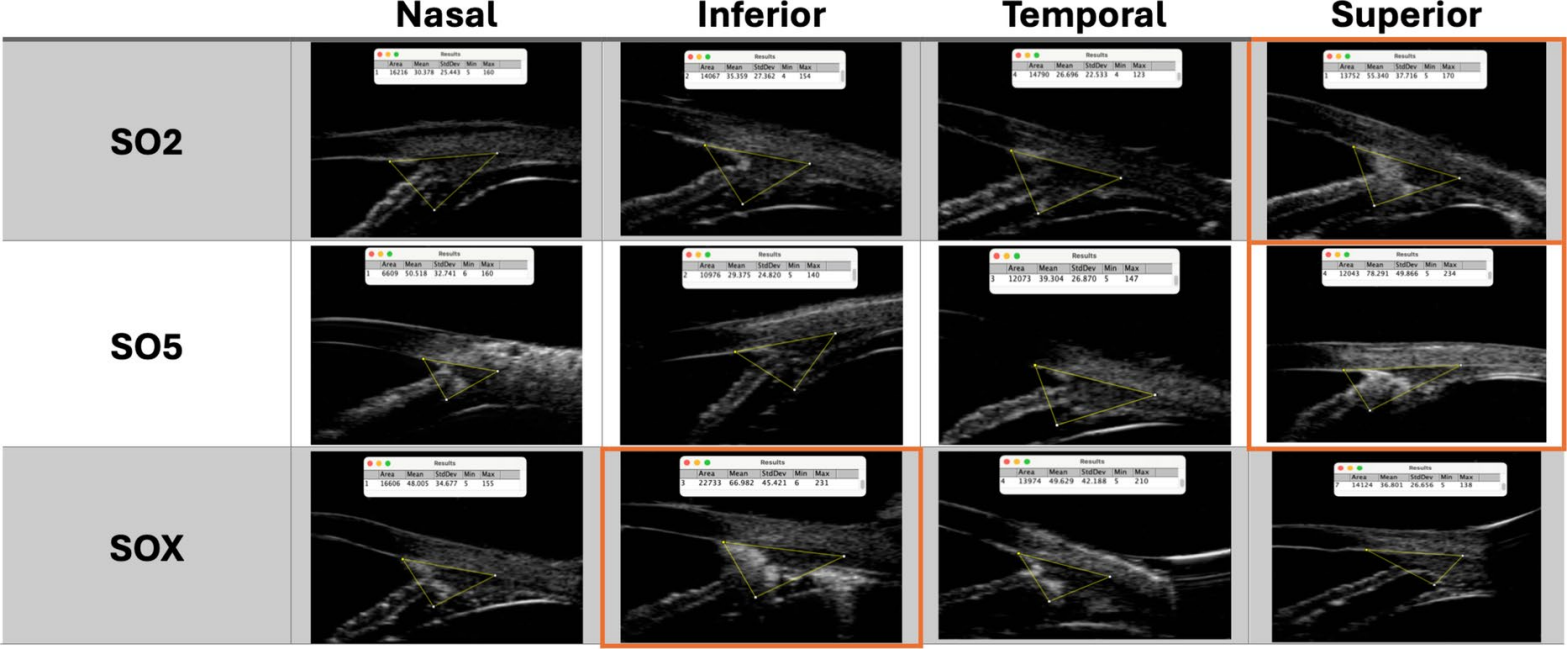
Composite of the four UBM radial scans tested in each study group (SO2 = Siluron 2000®; SO5 = RS-OIL ECS5000®; SOX = Densiron® XTRA). The “ImageJ” measurements obtained from the yellow Regions of Interest (ROI) areas are reported at the top of respective image. The superior quadrants for SO2 and SO5 group, and the inferior quadrant of SOX group are marked in orange

Regions of interest (ROI) were investigated with a 50 MHz transducer probe (ABSolu, Lumibird/Quantel Medical, France). The evaluation was conducted under topical anesthesia with the patient sitting under standard lighting (around 200 lx). All examinations were performed by the same trained examiner (FOA). Images were processed by a different author (CB) who was blind to whether the images belonged to the study or control cohort. We considered longitudinal images taken at the 3, 6, 9, and 12 o’clock positions of both eyes as representative of the respective AS quadrant (superior, S; inferior, I; nasal, N; and temporal, T; respecting the laterality). Four selected images per eye were exported in high-definition TIFF format with the contrast gain set at 90 dB, dynamic set at 40 dB, and time gain compensation set at 0 dB. The image was then processed using ImageJ (ver. 1.53 k NIH, USA). The ROI consisted of a right triangle outlined manually as follows (Fig. 1): after identifying Schwalbe’s line (SL, at the inner vertex of the V-shaped scleral hyperechogenicity), the first side connected SL to the medial apex of the pars plicata (PP). The second side was traced perpendicularly from the PP to the inner scleral margin. The third side joined this line to the SL. The resulting ROI included the trabecular meshwork, iris root, and anterior-most portion of the ciliary body, excluding the SOT-filled areas. The software analyzed the gray level of the pixels included in each ROI by returning the minimum and maximum values according to a standard scale ranging from 0 (lowest echogenicity = full black) to 255 (highest echogenicity = full white). Quantitative and topographical analyses were performed separately for the three tamponade groups and, in each one, for every tested quadrant. Figure 2 shows three representative study eyes, one for each SOT group, with the respective ImageJ values. In the quantitative analysis, the highest gray pixels recorded in the study eyes were averaged per quadrant and compared with the same parameter obtained in the corresponding quadrant of the control eyes. In the topographical analysis, any quadrant of each study eye was labelled as “involved” (YES/NO) when its gray maximum value exceeded the [average + 1 SD] value of the corresponding quadrant in the control cohort.

### Statistical analysis

Variables are expressed as the median and interquartile range (IQR), except for data from the ImageJ analysis, which are expressed as the mean ± SD. The normality of the distribution of quantitative variables was assessed using the Shapiro–Wilk test, together with skew and kurtosis indicators. The Mann–Whitney* U*-test was used to compare groups for quantitative data. The Kruskal–Wallis test was used to compare the duration of intraocular SOT among the three SOT groups. Adjustments for multiple comparisons were made using the Bonferroni correction. Spearman’s rank correlation was used to assess the relationship between the duration of intraocular tamponade and the number of quadrants involved in the study eyes. Statistical analyses were performed using SPSS (ver. 29.0.1.0; IBM., Armonk, NY, USA), and *P* < 0.05 was considered statistically significant.

## Results

Thirty-three patients (21 males, 12 females) met the inclusion criteria for both eyes (one with tamponade and the other as a control) and were included in the study (66 eyes). Fifteen eyes had high-density tamponade (SOX group), 11 eyes had PDMS 2000 cSt tamponade (SO2 group), and 7 eyes had PDMS 5000 cSt tamponade (SO5 group). At the UBM evaluation, none of the study eyes showed findings compatible with SOT bubbles in the anterior or posterior chamber, according to the evidence described by Grigera et al.^12^ This confirmed the reliability of the clinical selection of only those eyes without signs of SOT emulsification. Table 1 summarizes the demographics of the included patients, the pre-removal IOP, and the duration of intraocular tamponade in the study eyes separately for the three tamponade groups. At the time of UBM, 30/33 study eyes were being treated with hypotensive eyedrops (topical timolol maleate possibly combined with dorzolamide hydrochloride). The IOP (median with IQR) was 16.2 (13.5–19.0) mmHg in the SOX group, 20.5 (18.3–22.8) mmHg in SO2, and 10.8 mmHg (7.5–19.2) in SO5. These differences were not significant (*P* = 0.073). The duration of intraocular tamponade was 67 (range 49–75) days for the SOX group, 73 (50–138) for the SO2 group, and 78 (53 –233) for the SO5 group (intergroup *P* = 0.062).

**Table 1:** Demographics of the patients, pre-removal IOP and days of tamponade intraocular permanence in the study eyes (median with interquartile range)

	Age (years)	M/F	IOP (mmHg)	SOT (days)
SOX	66 (52–71)	7/8	16.2 (13.5–19)	67 (49–75)
SO2	60 (53–72)	8/3	20.5 (18.3–22.8)	73 (50–138)
SO5	57 (50–62)	6/1	10.8 (7.5–19.2)	78 (53–263)

M; males, F: females, IOP: intraocular pressure, SOT: silicone oil tamponade intraocular permanence, SOX: Densiron® Xtra group; SO2: Siluron2000® group; SO5: RS-OIL ECS5000® group

### UBM assessments and analyses

Keeping the three groups separate, Table 2 shows the mean ± SD of the highest gray values in each quadrant of the study eyes compared to the corresponding values in the control cohort. The following comparisons differed statistically: between the S quadrants in groups SO2 and SO5 (*P* = 0.04 and 0.02 respectively) and between the I quadrants in the SOX group (*P* < 0.01).

**Table 2:** Highest gray values (mean ± SD) per quadrant in the study eyes compared to the corresponding values in the control eyes

SO2 group			
Quadrant	Study eyes	Fellow eyes	*P* value
Nasal	136.4 ± 18.3	127.4 ± 26.9	0.423
Inferior	135.9 ± 14.1	127.8 ± 31.8	0.477
Temporal	129.7 ± 13.4	125.1 ± 41.7	0.722
Superior	168.2 ± 16.3	132.6 ± 10.6	0.041*

SO5 group			
Quadrants	Study eyes	Fellow eyes	*P* value
Nasal	130 ± 33.2	104.6 ± 15.5	0.063
Inferior	124.9 ± 7.7	121.4 ± 12.7	0.499
Temporal	132.3 ± 37.5	111.4 ± 2.8	0.091
Superior	169.1 ± 60.8	128.8 ± 11.3	0.018*

SOX group			
Quadrants	Study eyes	Fellow eyes	*P* value
Nasal	147.3 ± 47.4	134.9 ± 41.1	0.286
Inferior	190.1 ± 57.9	139.4 ± 1.4	0.001†
Temporal	150.5 ± 17.7	133.2 ± 12.1	0.221
Superior	138.7 ± 19.8	133.6 ± 14.1	0.629

*P < 0.05; †*P* < 0.01

Topographically, of the tissues included in the ROIs, the highest reflectivity areas were located mainly in the trabecular meshwork (64% of the involved quadrants). Table 3 details the topographic descriptive analysis in the study eyes. Concerning the number of “involved” quadrants, all of the SOX and SO5 eyes had at least one involved quadrant and only one eye in the SO2 group was free from any noticeable hyper-reflectivity. In the SOX group, 100% of the eyes had quadrant I involvement, while quadrant S was involved in 60%. In the SO2 group, quadrant S was involved in 73% and quadrant I in the 55% of the eyes. In the SO5 group, quadrant S was involved in 86% vs. 43% of quadrant I.

**Table 3:** Ratio of “involved” quadrants in the study eyes

	Nasal (%)	Inferior (%)	Temporal (%)	Superior (%)	Total (%)
SOX	9/15 (60)	15/15 (100)	8/15 (53)	9/15 (60)	41/60 (68)
SO2	6/11 (55)	6/11 (55)	5/11 (45)	8/11 (73)	25/44 (57)
SO5	5/7 (71)	3/7 (43)	5/7 (71)	6/7 (86)	19/28 (68)

SOX; Densiron® Xtra group, SO2; Siluron2000® group, SO5; RS-OIL ECS5000® group

i.e., in how many quadrants the gray maximum value exceeded the [average + 1 SD] value of the corresponding quadrant in the control cohort

The duration of intraocular SOT in the study eyes was positively correlated with the number of involved quadrants in the SO2 group (*P* < 0.01). No significant correlation was found between involved quadrants and pre-removal IOP.

## Discussion

The role of UBM in detecting abnormalities in eyes with in situ or already removed SOT fluid, regardless of the phakic state, has been known for over 20 years [16]. These changes, compared to normal eyes, may involve either the direct visualization of droplets floating in the AS or adjacent to its contour or increased echo-reflectivity in the filtering structures. The latter is attributed to direct SOT impregnation of the trabecular meshwork, iris root, or ciliary body, probably combined with localized inflammatory phenomena initiated by SOT-laden macrophages [14–17]. Although highly variable in size (micro to macro when > 2 μm in diameter), SOT bubbles from the vitreous chambers of treated eyes are thought to be responsible for the UBM findings and the possible related impairment of the aqueous outflow pathway [18].

The reported series included only eyes free from clinical signs of SOT emulsion in the AS, based on the international consensus scores detailed in the Methods. The study and control eyes were divided into three groups based on the SOT formulation and examined by UBM. For every included eye, four suitable longitudinal scans were captured, one per conventional circumferential trabecular quadrant. Finally, the exported images were processed using dedicated software and the resulting data analyzed.

Comparing the study and control cohorts, grouped according to the SOT formulation, the mean echo-reflectivity was significantly higher in the superior quadrant ROIs when the tamponade had a density < 1 (*i*.*e*., groups SO2 and SO5), and in the inferior quadrant for a density > 1 (SOX, see Table 2). For the SOX group, the findings were very significant. The correlation between the density of the SOT and the vertical localization of the hyper-reflective areas implies that the UBM findings are tamponade-related.

The topographical analysis showed UBM findings compatible with SO impregnation of the AS tissues in at least one sector in all but one of the study eyes, regardless of the duration or type of intraocular SOT. Since we included only eyes without clinically visible emulsification, it is remarkable that some impregnation of the draining structures occurs in virtually all eyes during SOT. The impregnation is mostly localized to the trabecular sectors contiguous with the SOT–aqueous interface when the patient’s head is raised. The correlation between the number of involved sectors and duration of tamponade was significant for group SO2. This lends relevance to the possible direct relationship between the duration of this specific SOT and the risk of secondary glaucoma. In our series, no other significant correlation was found. In particular, there was no correlation with male gender, as reported in a recent study of patients with similar clinical condition but with different ethnic characteristics and geographic locations [14] or with the preoperative IOP. However, in our study the IOP was controlled with hypotensive drugs when it exceeded the chosen threshold of 24 mmHg. This safety but arbitrary issue very likely altered or masked the relationship between the extension of the trabecular involvement and the natural IOP. We have planned to deepen the study of this issue by re-testing the study eyes of the present cohort after at least 6 months from the SOT removal, with particular attention to those eyes still requiring hypotensive medications.

The limitations of the present study mainly relate to the relatively limited number of eyes included in each SOT group. Moreover, although efforts were made to identify the ROI and hyper-reflective areas as objectively and precisely as possible, a degree of subjectivity remains in the image acquisition and processing system.

In conclusion, our study searched for SOT-related microstructural changes in the AS structures in eyes free from clinical evidence of SOT emulsification shortly before tamponade removal scheduled at the recommended time. Our ongoing study will evaluate the evolution of these abnormal echo-reflectivity findings after tamponade removal. This might further support the etiological role of the reported findings in the development of secondary glaucoma and provide further insights into the early pathogenesis of this feared complication.

## Data Availability

All data and material are available from the corresponding author.

## References

[1] Pa C, Becker B, Okun E, Canaan S (1962) The use of liquid silicone in retinal detachment surgery. Arch Ophthalmol 68:590–599. 10.1001/archopht.1962.00960030594005 14021325 10.1001/archopht.1962.00960030594005

[2] Sugar HS, Okamura ID (1976) Ocular findings six years after intravitreal silicone injection. Arch Ophthalmol 94:612–615. 10.1001/archopht.1976.03910030296009 1267641 10.1001/archopht.1976.03910030296009

[3] Federman JL, Schubert HD (1988) Complications associated with the use of silicone oil in 150 eyes after retina-vitreous surgery. Ophthalmology 95:870–876. 10.1016/s0161-6420(88)33080-0 3174036 10.1016/s0161-6420(88)33080-0

[4] Barr CC, Lai MY, Lean JS et al (1993) Postoperative intraocular pressure abnormalities in the silicone study. Silicone study report 4. Ophthalmology 100:1629–1635. 10.1016/s0161-6420(93)31425-9 8233387 10.1016/s0161-6420(93)31425-9

[5] Dresp JH, Menz DH (2005) Interaction of different ocular endotamponades as a risk factor for silicone oil emulsification. Retina 25:902–910. 10.1097/00006982-200510000-00014 16205571 10.1097/00006982-200510000-00014

[6] Honavar SG, Goyal M, Majji AB, Sen PK, Naduvilath T, Dandona L (1999) Glaucoma after pars plana vitrectomy and silicone oil injection for complicated retinal detachments. Ophthalmology 106:169–177. 10.1016/S0161-6420(99)90017-9 9917800 10.1016/S0161-6420(99)90017-9

[7] Azen SP, Scott IU, Flynn HW Jr et al (1998) Silicone oil in the repair of complex retinal detachments. A prospective observational multicenter study. Ophthalmology 105:1587–1597. 10.1016/S0161-6420(98)99023-6 9754162 10.1016/S0161-6420(98)99023-6

[8] Romano MR, Ferrara M, Nepita I et al (2021) Biocompatibility of intraocular liquid tamponade agents: an update. Eye (Lond) 35:2699–2713. 10.1038/s41433-021-01596-w 34035489 10.1038/s41433-021-01596-w PMC8452761

[9] Ichhpujani P, Jindal A, Jay Katz L (2009) Silicone oil induced glaucoma: a review. Graefe’s Archiv Clin Exper Ophthalmol 247:1585–1593. 10.1007/s00417-009-1155-x 10.1007/s00417-009-1155-x 19685070

[10] Bellucci C, Riboni N, Ricciotti G et al (2024) Contamination profile of different formulations of silicone oil tamponade before and after intraocular permanence for rhegmatogenous retinal detachment. Transl Vis Sci Technol 13:4. 10.1167/tvst.13.3.4 38466299 10.1167/tvst.13.3.4 PMC10929740

[11] Azzolini C, Pierro L, Codenotti M, Bandello F, Brancato R (1995) Ultrasound biomicroscopy following the intraocular use of silicone oil. Int Ophthalmol 19:191–195. 10.1007/BF00133737 8926132 10.1007/BF00133737

[12] Grigera DE, Zambrano A, Cazón GP, Cavanagh E, Girado SG (2000) Ultrasound biomicroscopy in silicone oil-filled eyes. Retina 20:524–531. 10.1097/00006982-200009000-00015 11039429 10.1097/00006982-200009000-00015

[13] Avitabile T, Bonfiglio V, Cicero A, Torrisi B, Reibaldi A (2002) Correlation between quantity of silicone oil emulsified in the anterior chamber and high pressure in vitrectomized eyes. Retina 22:443–448. 10.1097/00006982-200208000-00008 12172111 10.1097/00006982-200208000-00008

[14] Zhao H, Yu J, Zong Y, Jiang C, Zhu H, Xu G (2022) Characteristics of silicone oil emulsification after vitrectomy for rhegmatogenous retinal detachment: an ultrasound biomicroscopy study. Front Med 8:794786. 10.3389/fmed.2021.794786 10.3389/fmed.2021.794786 PMC8793062 35096885

[15] Romano MR, Ferrara M, Coco-Martin RM, Rickmann A, Spitzer MS, Steel DHW, Pastor JC (2023) Intraocular emulsion of silicone oil (items) grading system: an evidence-based expert-led consensus. Retina 43:1370–1376. 10.1097/IAE.0000000000003811 37071921 10.1097/IAE.0000000000003811

[16] Genovesi-Ebert F, Rizzo S, Chiellini S, Gabbriellini G, Laddaga F, Nardi M (1998) Ultrasound biomicroscopy in the assessment of secondary glaucoma after vitreoretinal surgery and silicone oil injection. Ophthalmologica 212(Suppl 1):4–5. 10.1159/000055409 9730735 10.1159/000055409

[17] Wickham LJ, Asaria RH, Alexander R, Luthert P, Charteris DG (2007) Immunopathology of intraocular silicone oil: retina and epiretinal membranes. Br J Ophthalmol 91:258–262. 10.1136/bjo.2006.103549 17005544 10.1136/bjo.2006.103549 PMC1857625

[18] Chan YK, Cheung N, Chan WS, Wong D (2015) Quantifying silicone oil emulsification in patients: are we only seeing the tip of the iceberg? Graefe’s Archiv Clin Exper Ophthalmol 253:1671–1675. 10.1007/s00417-014-2866-1 10.1007/s00417-014-2866-1 25418036

